# Sleep disturbances and behavioral symptoms in pediatric Sotos syndrome

**DOI:** 10.3389/fneur.2024.1360055

**Published:** 2024-02-16

**Authors:** Ilaria Frattale, Rachele Sarnataro, Martina Siracusano, Assia Riccioni, Cinzia Galasso, Massimiliano Valeriani, Giuseppina Conteduca, Domenico Coviello, Luigi Mazzone, Romina Moavero

**Affiliations:** ^1^Child Neurology and Psychiatry Unit, Department of Wellbeing of Mental and Neurological, Dental and Sensory Organ Health, Policlinico Tor Vergata Foundation Hospital, Rome, Italy; ^2^Department of Biomedicine and Prevention, University of Rome Tor Vergata, Rome, Italy; ^3^Systems Medicine Department, University of Rome Tor Vergata, Rome, Italy; ^4^Developmental Neurology, Bambino Gesù Children Hospital, IRCCS, Rome, Italy; ^5^Center for Sensory-Motor Interaction, Aalborg University, Aalborg, Denmark; ^6^Biotherapy Unit, IRCCS San Martino, Genoa, Italy; ^7^Laboratory of Human Genetics, IRCCS Istituto Giannina Gaslini, Genoa, Italy

**Keywords:** sleep, genetics, behavior, Sotos syndrome, children, pediatrics

## Abstract

**Background:**

Sotos syndrome (SoS) is a rare overgrowth genetic disease caused by intragenic mutations or microdeletions of the *NSD1* gene located on chromosome 5q35. SoS population might present cognitive impairment and a spectrum of behavioral characteristics, with a worse profile in patients with microdeletion. Although patients with SoS are known to have impaired sleep habits, very little data are available. The present study aimed to assess the prevalence of sleep disorders (SDs) in a pediatric cohort of patients with SoS and their correlation with neuropsychiatric profiles.

**Methods:**

We included patients with a SoS diagnosis and age < 18 years; all patients underwent a comprehensive neuropsychological assessment, including evaluation of cognition, adaptive functions through the Adaptive Behavior Assessment System-Second Edition (ABAS-II), and behavioral problems using the Achenbach Child Behavior Checklist (CBCL) and Conners’ Parent Rating Scale-Revised (CPRS-R:L) questionnaire. To investigate the presence of SD parents, the Sleep Disturbance Scale for Children (SDSC) was completed.

**Results:**

Thirty-eight patients (M 61%, F 39%, mean age 11.1 ± 4.65 years) were included in the study. Although only two had a prior SD diagnosis, 71.1% (N = 27) exhibited pathological scores on SDSC. No statistically significant associations were found between positive SDSC results and genetic microdeletion, intellectual disability (ID), or other medical conditions/treatments. However, a positive correlation emerged between SDSC scores and Conners’ Global Index (*p* = 0.048) and Restless/Impulsive (*p* = 0.01) scores, CBCL externalizing (*p* = 0.02), internalizing (p = 0.01), and total scores (*p* = 0.05). Conversely, a negative linear relationship was observed between the SDSC score and the ABAS GAC and ABAS CAD scores (*p* = 0.025).

**Conclusion:**

We detected an SD in 71.1% of our sample, with a positive relation between SD and internalizing and externalizing symptom levels, especially hyperactivity and impulsivity. Our study demonstrated a high prevalence of SD in pediatric patients with SoS, highlighting that all patients should be screened for this problem, which has a great impact on the quality of life of patients and their families.

## 1 Introduction

Sotos syndrome (SoS) is a rare overgrowth autosomal dominant syndrome with an incidence of approximately 1:14,000 live births (1). It is determined by intragenic mutations or microdeletions of the *NSD1* gene (nuclear receptor set domain-containing protein 1) located on chromosome 5q35 (2); in 95% of cases, it is caused by a *de novo* mutation (3). Recent studies have demonstrated that *NSD1* is involved in the MAPK/ERK pathway, an important regulator of cellular proliferation, differentiation, and apoptosis (4). The *NSD1* gene product is expressed in several organs, including the brain, skeletal muscle, white blood cells, lung, spleen, kidney, and thymus, and it has a role in the regulation of gene transcription.

The syndrome was described for the first time in 1964 by Juan Sotos (5), but diagnostic criteria (facial features, overgrowth, and developmental delay/learning disability) were defined only later in 1994 by Cole and Hughes (6). Considering the features observed in 266 patients with mutations in the *NSD1* gene, major characteristics, cardinals, and associated features were identified (Table 1).

**Table 1:** Clinical features of SoS.

Cardinal features >90%	Overgrowth (height and occipitofrontal circumference ≥ 2 standard deviations above mean values) Facial dysmorphisms like thin and elongated face, long chin, forehead prominent, dolichocephaly, sparse frontotemporal hair, and drooping palpebral fissure (7) Variable intellectual disability (8)
Major characteristics 15–90%	Behavioral problems Hypotonia Seizures Joint hyperlaxity Scoliosis Genitourinary anomalies Cardiac anomalies
Associated features <15%	Neuroendocrine tumors Hematological tumors Cataract Gastroesophageal reflux Conductive hearing loss Cryptorchidism Hemihypertrophy (3, 8–11)

SoS population presents a heterogeneous neuropsychological profile, with possible cognitive impairment of various degrees and a spectrum of different neuropsychiatric manifestations, including autism spectrum disorder (ASD), hyperactivity (12–19), oppositive symptoms (20), separation anxiety (21), and self- and heteroaggressiveness (22). A recent study conducted by our research team on 64 patients with SoS suggested that 5q35 microdeletion is associated with a higher severity of neuropsychiatric symptoms (including cognitive skills and behavioral issues) compared to mutation (23).

Patients with SoS also present impaired sleep habits. The first information about sleep disorders was reported in 1991 by Rutter (20), but only 30 years later this problem was further investigated (24), probably due to the rarity of the syndrome and the limited number of samples to be studied.

The present study aimed to assess the prevalence of sleep disorders in a cohort of children and adolescents with SoS and evaluate a possible relationship with the behavioral symptoms.

## 2 Materials and methods

The study involved patients who have been enrolled in the Child Neuropsychiatry Unit of Tor Vergata University Hospital of Rome. The population of this observational study is composed of SoS individuals included in a previous project conducted by our research team (23). The inclusion criteria of the present study were: a genetic diagnosis of SoS; age up to 18 years; and the availability to complete our questionnaires. In the context of our previous study (23), SoS patients underwent a complete neuropsychological standardized assessment, including cognitive intelligent quotient (IQ) evaluation through the non-verbal Leiter-R (Leiter international performance scale-revised), adaptive functioning (ABAS-II – Adaptive Behavior Assessment System-Second Edition), and parent-report behavioral questionnaire (CBCL, child behavior checklist; CPRS-R:L; Conners’ Parents Rating Scale-long form).

The Leiter-R is a standardized scale administered to children with communicational difficulties aged 2 to 20 years to evaluate their non-verbal cognitive abilities. The Leiter – R consists of 10 subtests: figure ground (FG), design analogies (DAs), form completion (FC), matching (M), sequential order (SO), repetitive pattern (RP), picture context (PC), classification (C), paper folding (PF), and figure rotation (FR), which make it possible to obtain a standardized non-verbal IQ score. In patients with more difficulties, it is possible to evaluate a brief IQ score through the FG, FC, So, and RP subtests (25).

The ABAS-II is a parent-report questionnaire available in two forms depending on the participant’s age: “0–5” and “5–21.” It provides information about the child’s adaptive functioning through the 241 parents’ answers for the “0–5″ form and 232 answers for the “5–21″ form, with scores ranging from 0 (“not able to”) to 3 (“able to do it and always performs it when needed”). The answers provide a way to define four skill domains: social (SAD), conceptual (CAD), practical (PAD), and General Adaptive Composite (GAC) scores deriving from the sum of other skill domains (26).

The typical score in the Leiter-R and ABAS-II is 100 ± 15 standard deviation, while the significant score is lower than 85.

The Achenbach Child Behavior Checklist (CBCL) is a parent-report questionnaire administrable in two forms depending on the child’s age: “18 months–5 years” and “6–18 years.” The answers provided by the parents allow us to obtain scores regarding behavioral problems and emotional symptoms using scores from 0 (not true) to 2 (often true). Considering the “18 months–5 years” form, the following areas are possible to detect: anxious/depressed, aggressive behavior, sleep problems, withdrawal, attention problems, emotional reactions, and somatic complaints. In the “6–18 years” form: withdrawn/depressed, social problems, somatic complaints, thought problems, aggressive behavior, rule-breaking behavior, anxious/depressed, and attention problems. Both forms have internalizing, externalizing, and total scores that can be considered unique scores for the statistical analysis. Considering the T-score, a behavior is considered borderline between 65 and 69, or significantly atypical, equal to or greater than 70 (27).

The Conners’ Parent Rating Scale-Revised (CPRS-R:L) is a parent-report questionnaire able to detect externalizing behavior constituted by 80 items organized in seven subscales (oppositional, social problems, anxious-shy, hyperactivity-impulsivity, perfectionism, and psychosomatic cognitive problems) (28). Total Conners’ Global Index (CGI), CGI Emotional Lability, CGI Restless-Impulsiveness, Diagnostic and Statistical Manual of Mental Disorders (DSM)-IV Total, DSM-IV Inattention, DSM-IV Hyperactive–Impulsive, and attention deficit hyperactivity disorder (ADHD) index scores are further detectable subscales (29). The T-score ranging from 61 to 69 is considered borderline, while it is significant if it is equal to or greater than 70 (28).

Parents were also asked to fill out an online survey that investigated medical comorbidities, with a focus on epilepsy and allergies, pharmacological treatment (including all neurological drugs and antihistaminics), rehabilitation therapy, instrumental examinations, and previous diagnoses of sleep disturbances.

Sleep quality was investigated through a thorough clinical history and the Sleep Disturbance Scale for Children (SDSC).

SDSC is a questionnaire validated by Bruni et al. in 1996 as a screening tool for pediatric sleep disorders. The answers to the 26 questions are converted into scores ranging from 1 to 5 depending on the frequency of the disturbance: 1 for never, 2 for occasionally, 3 for sometimes, 4 for often, and 5 for always (30).

The six most frequent types of disorder in adolescence and childhood are investigated: disorders in the initiation and maintenance of sleep (DIMS), sleep breathing disorders (SBDs), disorders of arousal (DA), sleep–wake transition disorders (SWTDs), disorders of excessive sleepiness (DOES), and night-time sweating (hyperhidrosis, SHY). The questionnaire is suggestive of sleep disturbances if the total score is equal to or greater than 39 and equal to or greater than 17 in the DIMS subscale, 7 in the SBD and SHY areas, 6 in DA, 14 in SWTD, and 13 in DOES.

Data from the clinical assessment and online survey were matched and included in a unique database for statistical analysis.

Demographical features considered for the purpose of the study were sex, age, intellectual disability (ID), genetic microdeletion, history and active epilepsy, other non-neurological comorbidities, antiseizure medication (ASM), other pharmacological treatments, and SD diagnosis.

An intellectual disability diagnosis was postulated considering the Leiter-R IQ score and the GAC score in the ABAS-II questionnaire.

The study protocol has been approved by the Ethical Committee of Tor Vergata University Hospital of Rome, Italy (#6022). All parents signed the informed consent form.

## 3 Results

The SDSC questionnaire was proposed to the parents of 64 SoS patients enrolled in our previous project (23), with a 60% response rate and therefore a total of 38 valid questionnaires. The mean age at completion was 11.1 ± 4.65 years. Male individuals were 60.5% (*n* = 23). The demographic and clinical characteristics of our sample are summarized in Table 2.

**Table 2:** Demographic and clinical characteristics of 38 participants.

	*N*	%
Total	38	
Mean Age	11.1 ± 4.65 years	
Female: Male	15:23	39: 61
Genetics (Microdeletion: Intramutation: Unavailable)	12: 24: 2	31.6: 63.2: 5.3
Epilepsy	10	26.3
Antiseizure medications	4	10.5
Other drugs	5	13.1
Other medical comorbidities *Allergies* *Orthopedic* *Genitourinary* *Gastrointestinal* *Eye* *Cardiac* *Orthodontic*	12 7 7 2 2 2 2 1	33 58.3 58.3 16.6 16.6 16.6 16.6 8.3
Rehabilitation	28	73.7
Sleep disorder diagnosis	2	5.3
Sleep disorder treatment	2	5.3

Microdeletion of the *NSD1* gene was detected in 31.6% (*n* = 12) of patients, while 63.2% (*n* = 24) presented an intragenic mutation. Genetic data were not available in 5.3% (*n* = 2).

Intellectual disability was diagnosed in 17 patients (44.7%). Ten patients had a history of epilepsy, of whom one had active epilepsy at the time of our evaluation (considering at least one seizure in the last year) and four were still on antiseizure medications. Among 12 individuals (31.6%), other non-neurologic medical conditions were present, with allergies (*n* = 7, 58.3%) being the most frequent one. Only two patients (5.3%) reported a previous diagnosis of sleep disorder.

### 3.1 Sleep disturbance scale for children

A score above the cutoff was found in 71.1% (*n* = 27), and the overall mean total score was 45.2 ± 12.3 (range 28–80).

As for the subscales, 6 (15.8%) patients in the DIMS scale, 10 (26.3%) in SBD, 4 (10.5%) in DA, 10 (26.3%) in SWTD, 2 (5.2%) in DOES, and 7 (18.4%) in SHY obtained a score above the cutoff (Supplementary Figure 1).

The mean reported sleep onset latency was 15 min, while the mean total sleep time was 8–9 h.

The chi-square test did not reveal a statistically significant association between a positive score in SDSC and genetic microdeletion (*p* = 0.63), sex (*p* = 1), ID (p = 1), epilepsy history (*p* = 0.7), active epilepsy (*p* = 0.3), ASM (*p* = 1), other drug intakes (*p* = 1), rehabilitation (*p* = 0.4), and other non-neurological comorbidities (*p* = 0.4).

### 3.2 Correlation of SDSC scores and behavioral symptoms

Twenty-nine patients (83%) achieved values above the cutoff in ABAS (mean score 68.6), 27 (84%) in CBCL (mean score 58.7), and 9 (26%) in Conners (mean score 61.6). The scores for all the subscales are reported in Table 3.

**Table 3:** Conners’, CBCL, and ABAS scores.

	Mean score	Median score	SD	Range
Conn_Oppositional	57.29	56	13.109	38–100
Conn_Cognitive problems	68.5	70	13.765	44–99
Conn_Hyperactivity	63.18	60	15.353	42–96
Conn_Anxious/Shy	59.91	56.5	13.666	41–88
Conn_Perfectionistic	53.64	51	10.694	36–84
Conn_Social Problems	68.2	64	19.043	42–99
Conn_Psychosomatic	51.12	48	9.943	39–82
Conn_ADHD Index	68.45	69	14.107	41–99
Conn_Restless/Impulsive	63.27	65	12.471	37–89
Conn_Emotional Lability	55.28	50.5	14.195	39–91
Conn_Global Index	61.16	62.5	13.274	38–91
Conn_DSM_IV (Inattentive)	65.35	67	13.289	41–98
Conn_DSM_IV_(Hyperactive–Impulsive)	61.73	61	12.751	42–90
Conn_DSM_IV_(ADHD total symptoms)	65.12	66	13.239	41–100
CBCL Externalizing_1_18	56.9	56.5	9.918	40–80
CBCL Internalizing_1_18	59.47	61	13.061	34–100
CBCL Total_1_18	59.72	62.5	10.142	34–75
ABAS_GAC_PC	66.09	63	20.914	40–120
ABAS_CAD_PC	71.29	67	20.423	43–120
ABAS_SAD_PC	72.03	70	17.070	50–120
ABAS_PAD_PC	65.09	58	21.861	40–113

IQ, intelligent quotient; Conn, Conners; ADHD, attention-deficit hyperactivity disorder; DSM, Diagnostic and Statistical Manual of Mental Disorders; CBCL, Achenbach Child Behavior Checklist; ABAS, Adaptive Behavior Assessment System; GAC, General Adaptive Composite; CAD, conceptual domain; SAD, social domain; PAD, practical domain.

The application of a two-tailed Student’s t-test for independent samples revealed no significant differences in mean scores of Conners, CBCL, and ABAS between subjects with and without an SD (Table 4).

**Table 4:** Two-tailed Student’s *t*-test for independent samples.

	SD	Mean	Mean Difference	Std. error difference	*T*	95% C.I.
IQ	0 1	83.29 74.08	9.202	11.369	*p* = 0.747	−14.050; 32.454
Conn_Oppositional	0 1	54.27 58.67	−4.394	4.784	*p* = 0.621	−14,128; 5.340
Conn_Cognitive problems	0 1	66.27 69.57	−3.292	5.091	*p* = 0.290	−13.663; 7.078
Conn_Hyperactivity	0 1	60.55 64.50	−3.955	5.716	*p* = 0.824	−15.613; 7.704
Conn_Anxious/Shy	0 1	60.73 59.52	1.206	5.083	*p* = 0.541	−9,148; 11,559
Conn_Perfectionistic	0 1	50.60 54.96	−4.357	4.041	*p* = 0.690	−12.597; 3.884
Conn_Social Problems	0 1	76.36 64.46	11.905	6.726	*p* = 0.409	−1.779; 25.589
Conn_Psychosomatic	0 1	50.00 51.54	−1.542	3.939	*p* = 0.848	−9.575; 6.492
Conn_ADHD Index	0 1	62.70 70.96	−8.257	5.223	*p* = 0.435	−18.908; 2.395
Conn_Restless/Impulsive	0 1	56.50 66.22	−9.717	4.471	*p* = 0.886	−18.835; −0.599
Conn_Emotional Lability	0 1	54.70 55.55	−0.845	5.501	*p* = 0.548	−12.080; 10.389
Conn_Global Index	0 1	55.30 63.82	−8.518	4.906	*p* = 0.393	−18.537; 1.500
Conn_DSM_IV (Inattentive)	0 1	62.73 66.61	−3.881	4.899	*p* = 0.754	−13,861; −6,098
Conn_DSM_IV_(Hyperactive–Impulsive)	0 1	59.18 63.00	−3.818	4.734	*p* = 0.781	−13.474; 5.838
Conn_DSM_IV_(ADHD total symptoms)	0 1	62.91 66.17	−3.265	4.894	*p* = 0.790	−13.234; 6.705
CBCL Internalizing_1_18	0 1	54.90 61.55	−6.645	4.916	*p* = 0.820	−16.685; 3.394
CBCL Externalizing_1_18	0 1	51.10 59.50	−8.400	3.526	*p* = 0.383	−15.601; −1.199
CBCL Total_1_18	0 1	55.60 61.59	−5.991	3.777	*p* = 0.702	−13.704; 1.722
ABAS_GAC_PC	0 1	71.55 63.58	7.962	7.604	*p* = 0.151	−7.508; 23.433
ABAS_CAD_PC	0 1	77.91 68.25	9.659	7.358	*p* = 0.259	−5.312; 24.630
ABAS_SAD_PC	0 1	72.91 71.63	1.284	6.305	*p* = 0.105	−11.543; 14.111
ABAS_PAD_PC	0 1	67.82 63.83	3.985	8.050	*p* = 0.171	−12.392; 20.362

IQ, intelligent quotient; Conn, Conners; ADHD, attention-deficit hyperactivity disorder; DSM, Diagnostic and Statistical Manual of Mental Disorders; CBCL, Achenbach Child Behavior Checklist; ABAS, Adaptive Behavior Assessment System; GAC, General Adaptive Composite; CAD, conceptual domain; SAD, social domain; PAD, practical domain.

Linear regression reveals a positive relationship between the SDSC scores and Conners’ and CBCL subscales regarding externalizing and internalizing symptoms (Conners’ CGI Total and Conners’ restless/impulsive, CBCL externalizing, internalizing, and total score). This indicates that an increase in these values is associated with an increased SDSC score. Furthermore, there is a negative linear relationship between the SDSC score and ABAS subscales.

In the logistic regression model including IQ, Conners’, CBCL’s, and ABAS’s subscale independent variables, none of them appeared to be a significant risk factor for positive SDSC. All the statistical linear regression test scores and logistic regression model scores are reported in Table 5.

**Table 5:** Linear regression analysis and logistic regression model.

	Linear regression analysis			Logistic regression	
	Adj. *R*^2^	Standardized coefficients	Sig.	Exp (B) odd ratio	Sig.
IQ	−0.032	0.049	*p* = 0.794	0.986	*p* = 0.413
Conn_Oppositional	0.020	0.220	*p* = 0.204	1.030	*p* = 0.359
Conn_Cognitive problems	0.000	0.173	*p* = 0.328	1.018	*p* = 0.510
Conn_Hyperactivity	0.003	0.185	*p* = 0.303	1.018	*p* = 0.482
Conn_Anxious/Shy	−0.026	−0.070	*p* = 0.694	0.993	*p* = 0.807
Conn_Perfectionistic	0.013	0.208	*p* = 0.245	1.045	*p* = 0.284
Conn_Social Problems	−0.029	−0.035	*p* = 0.840	0.966	*p* = 0.091
Conn_Psychosomatic	−0.024	0.091	*p* = 0.615	1.017	*p* = 0.688
Conn_ADHD Index	0.048	0.279	*p* = 0.116	1.047	*p* = 0.129
Conn_Restless/Impulsive	**0.146**	**0.415**	***p* = 0.016**	1.074	*p* = 0.049
Conn_Emotional Lability	−0.028	0.073	*p* = 0.691	1.004	*p* = 0.874
Conn_Global Index	**0.095**	**0.352**	***p* = 0.048**	1.057	*p* = 0.100
Conn_DSM_IV (Inattentive)	−0.001	0.172	*p* = 0.329	1.024	*p* = 0.423
Conn_DSM_IV_(Hyperactive–Impulsive)	0.002	0.183	*p* = 0.307	1.025	*p* = 0.414
Conn_DSM_IV_(ADHD total symptoms)	−0.001	0.173	*p* = 0.329	1.020	*p* = 0.498
CBCL Externalizing_1_18	**0.244**	**0.518**	***p* = 0.02**	1.118	*p* = 0.037
CBCL Internalizing_1_18	**0.274**	**0.546**	***p* = 0.01**	1.047	*p* = 0.186
CBCL Total_1_18	**0.207**	**0.483**	***p* = 0.05**	1.063	*p* = 0.130
ABAS_GAC_PC	**0.118**	**−0.379**	***p* = 0.025**	0.982	*p* = 0.298
ABAS_CAD_PC	**0.118**	**−0.379**	***p* = 0.025**	0.977	*p* = 0.198
ABAS_SAD_PC	0.069	−0.310	*p* = 0.07	0.996	*p* = 0.834
ABAS_PAD_PC	0.072	−0.315	*p* = 0.07	0.992	*p* = s0.613

IQ, intelligent quotient; Conn, Conners; ADHD, attention-deficit hyperactivity disorder; DSM, Diagnostic and Statistical Manual of Mental Disorders; CBCL, Achenbach Child Behavior Checklist; ABAS, Adaptive Behavior Assessment System; GAC, General Adaptive Composite; CAD, conceptual domain; SAD, social domain; PAD, practical domain.

For the linear regression analysis, the statistically significant scores are highlighted in bold font: Conn_Restless/Impulsive *p* = 0.016; Conn_Global Index *p* = 0.048; CBCL Externalizing_1_18 *p* = 0.02; CBCL Internalizing_1_18 *p* = 0.01; CBCL Total_1_18 *p* = 0.05; ABAS_GAC_PC *p* = 0.025; ABAS_CAD_PC *p* = 0.025.

The logistic regression model shows no statistical probability of having SD considering IQ, Conners’, CBCL, and ABAS subscales.

## 4 Discussion

In our sample, we were able to detect sleep disorders in 71.1% of patients through a comprehensive collection of detailed clinical histories and the administration of the SDSC. This finding demonstrates that these disorders are much more prevalent in children and adolescents with SoS than in the general pediatric population, where the prevalence is 20–30% (31–33).

The most commonly identified sleep problems were SBD and SWTD, both occurring in 26.3%, possibly linked to the high incidence of congenital malformations of the neck and head in this population, such as general hypotonia, including weakness in the muscles of swallowing, macroglossia, high arched palates, alveolar cleft and cleft lip, and palate, which could contribute to respiratory and feeding difficulties (34). Regarding the other subscales, we observed a score above the cutoff for SHY at 18.4% and for DIMS at 15.8%.

In our sample, SD is not significantly associated with genetic microdeletion, ID, or other medical conditions and treatments.

However, the SDSC score is related to the level of internalizing and externalizing symptoms, particularly in the hyperactivity and impulsivity domains. According to our findings, better adaptive functioning is correlated with lower SDSC scores. This is noteworthy despite all the adaptive functioning scores identified with ABAS-II being significantly pathological, indicating an adaptive functioning level below the normal range.

Behavioral problems within the SoS spectrum are among the major characteristics, with a prevalence ranging from 15 to 90%, depending on the examined study group. It is important to note that, being a rare syndrome, literature data often refer to case reports or small studies.

In 1991, Rutter first described sleep problems related to early awakenings in a small sample of 11 SoS subjects (20). In a recent study, 40 patients with a mean age of 11 years (1.3–30 years) and genetic, possible, and probable diagnoses of SoS were compared with data from healthy subjects in the literature. The study showed increased resistance at bedtime, anxiety surrounding sleep, nocturnal awakenings, parasomnias, daytime sleepiness, and evidence of sleep-disordered breathing (SDB). The authors compared their population with one presenting intellectual and developmental disabilities, and they found that patients with SoS more frequently presented SBDs and parasomnias (24). These studies have certain limitations. Rutter (20) employed a non-standardized parent report. In the study conducted by Stafford et al. (24), participants with probable, possible, and genetically diagnosed SoS were included. They completed the Children’s Sleep Habits Questionnaire (CHSQ), validated for children aged 4 to 10 years, to establish a standardized parameter for all included patients, regardless of age (1.3 to 30 years old).

Sleep issues are prevalent across all stages of childhood, encompassing infants, toddlers, preschoolers, school-age children, and adolescents. During children’s brain development, sleep plays a key role, necessary for typical synaptic development and maturation of brain areas (35). Poor sleep can have significant repercussions on the cognitive side, and, due to the involvement of the prefrontal cortex, it can also lead to alterations in executive functions (36–38). It is also associated with behavioral disturbances, particularly a tendency toward externalizing disorders (39), irritability, and difficulty modulating emotions and impulses (39–41). Several studies have demonstrated the correlation between good sleep and a positive impact on future behavioral and socioemotional aspects lasting into adulthood (39, 42, 43). Chronic poor sleep habits, particularly short sleep durations, can also increase the risk of obesity and metabolic diseases (44–46). Sleep-related breathing disorders, ranging from habitual snoring to obstructive sleep apnea syndrome, can significantly affect emotional intelligence, cardiovascular functions, orofacial thrive, and the neuroendocrine and central nervous systems (47).

Furthermore, studies have shown that caregivers of patients with sleep disturbances experience high levels of distress (48–50), and stress levels improve when the child’s sleep habits improve (51, 52).

Therefore, early identification of an SD is crucial to prevent repercussions on the cognitive, emotional, and behavioral spheres, especially in a population that is genetically predisposed. It is essential to support families in managing this condition. Clinicians should specifically evaluate this aspect, as in our population, only two patients have already had a diagnosis of SD.

Our study does have some limitations. Not all patients responded to our questionnaires, and it is likely that parents of those with sleep disturbances were more likely to respond, potentially influencing the final rate of SD detected in our population. Moreover, a comprehensive physical examination of all study participants to correlate with the high prevalence of sleep disorders in SBD and SWTD areas was not feasible. Nonetheless, it is well established that the SoS phenotype encompasses head and neck malformations.

## 5 Conclusion

To the best of our knowledge, this is the first study attempting to correlate the presence of sleep disorders with behavioral symptoms in the pediatric SoS population. Internalizing and externalizing symptoms, especially in the hyperactivity and impulsivity domains, are directly related to more severe sleep symptoms, whereas typical adaptive behaviors are associated with milder sleep problems. Early detection of sleep disorders is crucial for enabling prompt treatment, reducing the onset of internalizing and externalizing problems, and preventing adverse impacts on the quality of life for caregivers.

Additionally, addressing behavioral symptoms may positively impact sleep, given the bidirectional connection between the two.

The high prevalence of SD in our population underscores the need to include an investigation of sleep habits and quality in the clinical evaluations of these patients to identify and manage them correctly.

## Data availability statement

The original contributions presented in the study are included in the article/supplementary material, further inquiries can be directed to the corresponding author.

## Ethics statement

The studies involving humans were approved by Ethical Committee of Tor Vergata University Hospital of Rome, Italy. The studies were conducted in accordance with the local legislation and institutional requirements. Written informed consent for participation was not required from the participants or the participants’ legal guardians/next of kin in accordance with the national legislation and institutional requirements. Written informed consent was obtained from the minor(s)’ legal guardian/next of kin for the publication of any potentially identifiable images or data included in this article.

## Author contributions

IF: Data curation, Formal analysis, Writing – original draft. RS: Investigation, Writing – review & editing. MS: Writing – review & editing. AR: Writing – review & editing. CG: Writing – review & editing. MV: Writing – review & editing. GC: Writing – review & editing. DC: Writing – review & editing. LM: Writing – review & editing. RM: Conceptualization, Data curation, Formal analysis, Supervision, Writing – original draft.
