# Supplementary material for: Sleep disturbances and behavioral symptoms in pediatric Sotos syndrome
Source: Front Neurol. 2024 Feb 16;15:1360055. doi: 10.3389/fneur.2024.1360055 (PMC10904657; doi:10.3389/fneur.2024.1360055)
Supplement: Supplementary file 1 [file Data_Sheet_1.PDF]

Supplementary Material

1.1 Supplementary Figures

Fig.1

Pathological score in SDSC

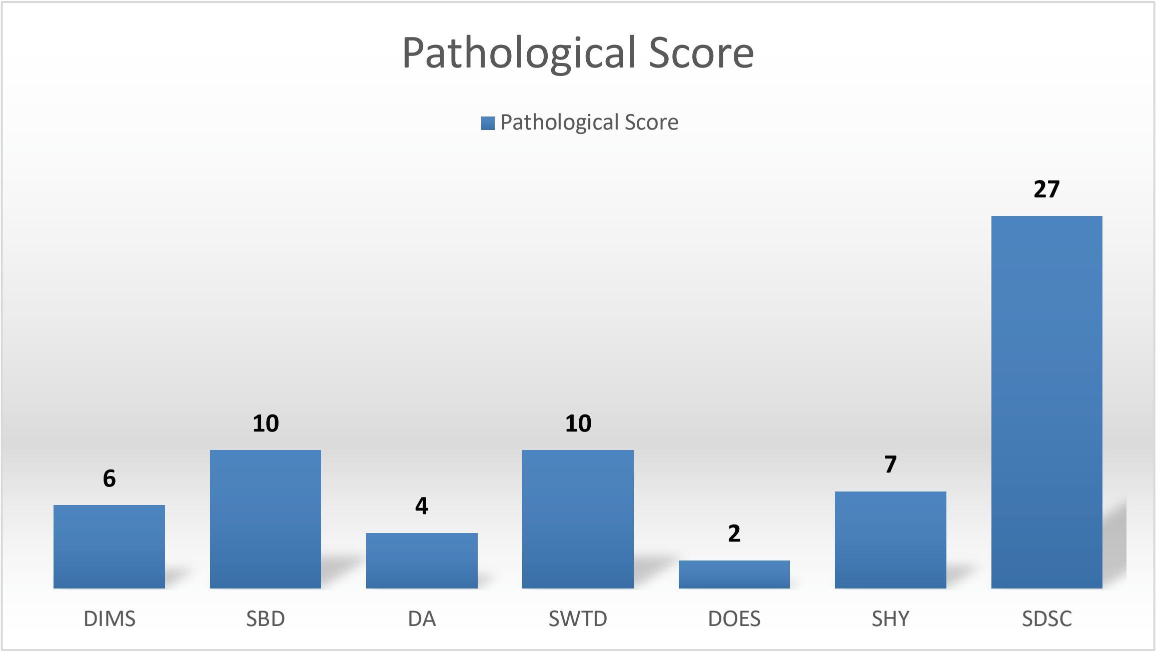

Fig.1

Pathological score in SDSC (total score and subscales) in our SoS sample

Abbreviation: DIMS Difficulties in Initiating and Maintaining Sleep; SBD Sleep relating Breathing Disorders; DA Disorders of Arousal; SWTD Sleep-Wake Transition Disorder; DOES Disorders Of Excessive Somnolence; SHY Sleep related HYperhydrosis.
